# Biofilm Formation Ability and Presence of Adhesion Genes among Coagulase-Negative and Coagulase-Positive Staphylococci Isolates from Raw Cow’s Milk

**DOI:** 10.3390/pathogens9080654

**Published:** 2020-08-14

**Authors:** Joanna Gajewska, Wioleta Chajęcka-Wierzchowska

**Affiliations:** Department of Industrial and Food Microbiology, University of Warmia and Mazury in Olsztyn, Plac Cieszyński 1, 10-726 Olsztyn, Poland; wioleta.chajecka@uwm.edu.pl

**Keywords:** biofilm, virulence factors, *S. aureus*, coagulase-negative staphylococci, raw milk

## Abstract

The capacity for biofilm formation is one of the crucial factors of staphylococcal virulence. The occurrence of biofilm-forming staphylococci in raw milk may result in disturbances in technological processes in dairy factories as well as the contamination of finished food products. Therefore, this study aimed to determine the prevalence and characteristics of staphylococcal biofilm formation in raw milk samples and to explore the genetic background associated with biofilm formation in those isolates. The material subjected to testing included 30 cow’s milk samples acquired from farms in the central part of Poland. A total of 54 staphylococcal strains were isolated from the samples, of which 42 were classified as coagulase-negative (CoNS) staphylococci belonging to the following species: *S. haemolyticus*, *S. simulans*, *S. warneri*, *S. chromogenes*, *S. hominis*, *S. sciuri*, *S. capitis*, *S. xylosus* and *S. saprophyticus*, while 12 were classified as *S. aureus*. The study examined the isolates’ capacity for biofilm formation and the staphylococcal capacity for slime production and determined the presence of genetic determinants responsible for biofilm formation, i.e., the *ica*A, *ica*D, *bap* and *eno* and, additionally, among coagulase-negative staphylococci, i.e., the *aap*, *bhp*, *fbe*, *emb*P and *atl*E. Each tested isolate exhibited the capacity for biofilm formation, of which most of them (79.6%) were capable of forming a strong biofilm, while 5.6% formed a moderate biofilm, and 14.8% a weak biofilm. A capacity for slime production was demonstrated in 51.9% isolates. Most of the tested staphylococcal strains (90.7%) had at least one of the tested genes. Nearly half (47.6%) of the CoNS had the *eno* gene, while for *S. aureus*, the *eno* gene was demonstrated in 58.3% isolates. The frequency of the *bap* gene occurrence was 23.8% and 25% in CoNS strains and *S. aureus*, respectively. The *fbe* gene was demonstrated in only three CoNS isolates. The presence of the *ica*A was only demonstrated in CoNS strains (24.1%), while the *ica*D was found in both CoNS strains (21.4%) and *S. aureus* (100%). Among the CoNS, the presence of the *emb*P (16.7%), *aap* (28.6%) and *atl*E (23.8%) was demonstrated as well. The obtained study results indicate that bacteria of the *Staphylococcus* spp. genus have a strong potential to form a biofilm, which may pose a hazard to consumer health.

## 1. Introduction

Biofilm is a bacterial structure that covers production surfaces, usually combined with mineral and organic impurities. Bacterial biofilm present protective functions against dehydration, bacteriophage infection and antimicrobial agents. Importantly, the biofilms are a major concern in the food industry for their potential to resist antimicrobial treatments, facilitating pathogenic contamination and food degradation [1].

The quality of dairy products is closely linked to the quality of raw milk which, in turn, is determined by numerous environmental factors, including the cows’ microflora, the quality of milking equipment, the quality of barn air, the employees’ hygiene, the cleanliness of storage tanks and processing equipment (e.g., centrifuges or pasteurizers) [2,3]. Raw milk, due to the presence of protein, fat, glucose or mineral salts, is an ideal environment for the growth of many microorganism groups [4]. The quality of raw milk affects production efficiency and contributes to the occurrence of sensory defects in finished products, e.g., rancidification [5]. The contamination of milk is significantly contributed to poor hygienic practices, animal living conditions and health status and improper raw material storage. Cows’ teats are a potential source of microbiological contamination of raw milk. They may be a source of many microorganism groups that may vary from one farm to another [6]. The absence of production hygiene may result in the formation of a biofilm on milking equipment, from which the microorganisms may be released to the raw material [7]. 

Since bacteria of the *Staphylococcus* genus are a widespread group of microorganisms found in both human and animal environments, they are a significant part of the raw milk microflora concerned. One of the major foodborne pathogens is *S. aureus*; however, recent years have seen an increased interest in coagulase-negative staphylococci (CoNS) which are currently classified as opportunistic pathogens [8]. The staphylococcal capacity for biofilm formation is one of the most important processes contributing to an increase in resistance to adverse environmental conditions and its colonization. Staphylococci can form biofilm alone, but more often co-form a biofilm with other groups of microorganisms, including pathogenic bacteria, e.g., *Listeria monocytogenes*, *Bacillus cereus*, *Pseudomonas aeruginosa* [9,10]. It was demonstrated that staphylococci have the potential for biofilm formation on surfaces made of stainless steel, rubber and plastics which are found in milking equipment, storage tanks and production lines [11]. The occurrence of biofilms in the dairy sector may cause economic losses due to the impaired quality/performance, the spoilage of food, problems with food safety and difficulties in maintaining the basic cleanliness of machinery and equipment [12].

Biofilm formation is a multi-stage process involving the adhesion, accumulation, maturation and dispersion of the biofilm [10]. The capacity for biofilm formation is determined by many genetic factors. The main biofilm component is the Polysaccharide Intercellular Adhesin (PIA) molecule synthesized by enzymes encoded by the genes *ica*A, *ica*B, *ica*C, *ica*D, that form the *ica*ABCD operon [13]. In the process of biofilm formation by staphylococci, various adhesin types participate. These include fimbria-like proteins SSP-1 and SSP-2 (Staphylococcal Surface Proteins), AtlE adhesin (autolysin E), *aae* adhesin (autolysin/adhesin Aae) or the fibrinogen-binding protein [14]. Cucarella et al. [15] in a study on the biofilm of *S. aureus* strains isolated from cows affected by mastitis, also indicated other *bap* protein (biofilm-associated protein) encoded by the *bap* gene, which promotes both the initial adhesion of strains to abiotic surfaces as well as the intercellular adhesion. Staphylococcal adhesins belong collectively to a group called MSCRAMM for short (Microbial Surface Components Recognising Adhesive Matrix Molecules) [16]. The composition of MSCRAMM includes fibronectin-binding proteins *fnb*A and *fnb*B (fibrinogen-binding proteins A and B), *clf*A and *clf*B fibrinogen-binding proteins (clumping factors A and B), cell wall proteins (type 5 and 8 Capsule) and collagen-binding proteins, *cna*, and fibrinogen-binding proteins, *fib*, which are capable of binding with various extracellular mammalian proteins and with abiotic surfaces. The ability of coagulase-positive and coagulase-negative staphylococci to adhere to various surfaces is also determined by the *eno* gene (laminin-binding protein) that encodes the laminin-binding protein [17]. In coagulase-negative staphylococci, the first stage of biofilm formation takes place via the proteins present in the bacterial cell wall, i.e., the *bhp* (bap homologous protein), *atl*E (autolysin E), and *fbe* (fibrinogen binding protein) [18]. The accumulation stage is characterized by the formation of polysaccharide intercellular adhesin (PIA) which is encoded by the proteins *ica*A, *ica*B, *ica*C and *ica*D that form the *ica*ABCD operon. The data also noted the formation of a PIA-independent biofilm by CoNS. In this case, in the formation of a biofilm, a significant role is played by the *aap* (accumulation-associated protein) proteins (extracellular matrix biofilm protein) [19].

This study aimed to assess the capacity of strains belonging to the *Staphylococcus* genus isolated from raw cow’s milk for biofilm formation, slime-production capacities and identify genetic determinants associated with the biofilm.

## 2. Results

Fifty-four staphylococci were isolated from 30 samples of raw cow milk obtained from central Poland milk farms. All samples were positive for staphylococci. From 54 staphylococci strains 42 were classified as CoNS. The identification of matrix-assisted laser desorption and ionization (MALDI) allowed us to classify 11 (20.4%) as *S. haemolyticus*, eight (14.8%) as *S. simulans*, five (9.3%) as *S. warneri*, four (7.4%) as *S. chromogenes*, three (5.6%) as *S. hominis*, two (3.7%) as *S. sciuri* and single isolates belonged to the species *S. capitis*, *S. xylosus*, *S. saprophyticus*, 12 strains (22.2%) were classified as *S. aureus*. All of the isolates (*S. aureus* and CoNS) were able to form a biofilm. The majority of the strains (79.6%) were capable of producing a strong biofilm, followed by 5.6% which produced a moderate biofilm and 14.8% which produced a weak biofilm. The ability to form a strong biofilm was more often observed in CoNS than *S. aureus* (85.7% vs. 58.3%). It is worth noting that no isolate was characterized by a lack of biofilm production ability. 

Slime production was investigated as a possible major determinant of bacterial adherence to biotic and abiotic surfaces. In the Congo Red Agar method, as many as 28 of all strains (51.9%) of *Staphylococcus* grew in the form of black colonies, which was assumed to be slime producing strains. In the form of bordeaux colonies, 20 (37%) of the tested isolates grew, while red colonies were observed in six (11.1%) strains. In view of the assumptions, these strains were classified as not producing slime (Table 1).

The majority of *S. aureus* isolates (80%) producing slime were also able to form a strong biofilm. Different results were obtained for CoNS strains, where both strains with and without slime producing showed the ability to produce strong biofilm (87% and 84.2% respectively). Based on statistical analysis, there was no correlation between biofilm production ability and slime production capacity in both *S. aureus* and CoNS (r = −0.174). 

The majority of all staphylococcal strains (90.7%) had at least one of the tested genes. Among CoNS, 31 strong, two moderate and four weak biofilm-forming isolates were found to have single or multiple biofilm-associated genes. Five CoNS strains from the genus *S. chromogenes*, *S. haemolyticus*, *S. hominis*, *S. xylosus* and *S. simulans* despite their ability to produce a strong biofilm, did not have any genetic determinants associated with the biofilm. Almost half (47.6%) of CoNS isolates had the *eno* gene and (58.3%) *S. aureus* strains. The frequency of the *bap* gene was 23.8% and 25%, respectively in CoNS strains and *S. aureus*. The *fbe* gene was found only in three CoNS strains producing a strong biofilm (7.1%) from the genus *S. epidermidis* and *S. haemolyticus* strains. None of the biofilm-forming isolates were positive for the *bhp* gene. From all of the tested staphylococci strains, less than 25% (24.1%) showed the presence of the *ica*A gene of which it was present only in CoNS (n = 13). A higher percentage (38.9%) of them had the *ica*D gene, which was found in both CoNS (21.4%) and all *S. aureus* strains (100%). The *emb*P gene was found in seven (16.7%) CoNS isolates. Biofilm-forming CoNS isolates also possessed *aap* gene (28.6%) and *atl*E gene (23.8%). No CoNS isolates possessed all nine genes (Table 2).

The association between the phenotypic MTP method and genetic determinants responsible for biofilm production ability is presented in Table 3. Taking into account the overall biofilm production ability of *S. aureus* strains, based on a statistical analysis no correlation between the presence of *ica*A, *bap*, *eno* and biofilm production ability was demonstrated (correlation coefficient respectively 0.02; −0.05; −0,04). Nevertheless, a significant correlation between biofilm production capacity and the presence of *ica*D (r = 1) was confirmed. For CoNS isolates, statistical analysis showed no correlation between the presence of the tested genes and biofilm production (correlation coefficient < 0.2).

## 3. Discussion

Raw milk sourced even from healthy cows always contains small amounts of microorganisms. However, failure to observe milking hygiene or the improper storage of raw material may result in a several-fold increase in the bacterial count in 1 mL [20]. The obtained study results indicate that raw milk originating from healthy cows is a source of the *Staphylococcus* genus, which can form a biofilm. The adverse changes resulting from the formation of a biofilm are the cause of severe losses in the economy as well as the spread of difficult-to-treat infections. Since biofilm bacteria show much greater resistance to antibiotics than their free-living counterparts, they pose a significant risk under hospital conditions (the colonization of catheters, implants or valves) [21]. In the food industry, the presence of microorganisms capable of forming a biofilm is also extremely significant. A microbiological biofilm formed on the surfaces of machines and equipment in food processing plants not only adversely affects the safety and quality of a finished product but, more importantly, poses a hazard to consumer health as the biofilm structure may contain pathogenic bacteria [9,22]. A biofilm formed on equipment surfaces has an adverse effect on the course of technological processes inter alia by inhibiting the heat exchange processes, blocking the liquid flow, increased pressure drops or the corrosion of metal surfaces, all of them which result in considerable economic losses [23]. A complex and multi-layered biofilm structure results in resistance to the action of disinfectants during the processes of technological line washing and disinfection, which may cause the secondary contamination of food products [11]. A study on bacterial resistance to the action of disinfectants showed that the strains making up the biofilm structure are almost 1000 times more resistant to the action of washing agents than the bacteria living freely in a suspension [7].

The research carried out to date, concerning the capacity for biofilm formation of staphylococci isolated from raw milk mainly concentrated on *S. aureus* strains which are the main etiological factor causing clinical or sub-clinical mastitis. However, few studies have addressed the issue of coagulase-negative staphylococcal strains in the context of their capacity for biofilm formation [23,24]. Since CoNS are part of normal microflora and are found on healthy udder skin or on farmers’ hands, they are often the microflora isolated from milk samples in many countries [25]. Despite the numerous doubts about the effects of CoNS on ruminant adder health, these strains are increasingly often recognized as a significant cause of this condition in cows worldwide [23,24,26]. Although the authors suggest that the biofilms formed by CoNS do not contribute to an increase in mastitis level, they serve an important role in the survival of these microorganisms in the dairy production environment [23,26]. It is worth noting that the presence of potential pathogenic agents in certain strains of coagulase-negative staphylococci (the production of enterotoxins or biogenic amines) has a significant effect on food safety [27]. The obtained results of both this study and studies by other authors indicate a higher frequency of occurrence of CoNS than *S. aureus* isolates in raw milk [28,29].

Of the numerous methods for determining microorganisms’ capacity for biofilm formation, the most frequently applied is the microtitration plate (MTP) method [30,31]. All isolates subjected to testing were capable of biofilm formation in a strong, moderate or weak manner. The results of this study and studies by other authors [29,32,33,34] indicate a high percentage of staphylococci isolated from milk and the dairy production environment, which are capable of forming a biofilm. More importantly, a higher percentage of CoNS strains exhibited the capacity for forming a strong biofilm, compared to *S. aureus* strains. Similar observations were also demonstrated by other authors [29,35], which indicates the great potential for biofilm-forming by CoNS strains. The occurrence of microorganisms capable of biofilm formation suggests the need for the introduction of stricter control of milking hygiene, raw material storage and technological lines to prevent the spread of biofilm-forming staphylococcal strains which may result in disturbances in technological processes as well as the contamination of finished food products. 

The capacity for slime production was determined based on the morphology of colonies which had grown on the Congo Red Agar medium. More than 50% of all the tested isolates were qualified as slime-forming isolates. Darwish et al. [33] demonstrated that a much higher percentage of staphylococci were capable of secreting slime. A study of isolates originating from mastitis-affected milk was carried out by Melo et al. [34]. The authors demonstrated a higher percentage of slime-producing strains (85%) compared to the results of this study. The discrepancies in the results obtained, which indicate the capacity for slime secretion, may result from a different source of strains (milk from healthy cows and cows with mastitis) and also a different interpretation of the colony morphology on the CRA medium. In this study, the growth of strains in the form of black colonies was assumed to be a positive result, while the colonies in claret or red colour were assumed to be negative. It should be noted, however, that various researchers classify strains differently in terms of morphology. Some of them classified the claret-coloured colonies as moderate slime producers.

The *ica* operon, which encodes the PIA, is regarded by many researchers to be a marker of *Staphylococcus* genus strain pathogenicity. However, other researchers have found the presence of these proteins in CoNS which are not capable of forming a biofilm [36]. The *ica*A gene was present exclusively among the coagulase-negative strains (31%), while the *ica*D was present in all *S. aureus* isolates and in 21.4% of CoNS strains, which was not correlated with the results for biofilm-forming capacity obtained by the phenotypic method. A low percentage of CoNS isolates with *ica*A was also noted by Srednik et al. [37], (who demonstrated the presence of this protein exclusively among 10% of the tested isolates) and by Osman et al. [23] (13.8%). The presence of *ica*D among CoNS was demonstrated by a higher percentage (76.6%) by Osman et al. [23] and Darwish et al. (47.1%) [33]. As regards the *S. aureus* isolates, the obtained results for the frequency of occurrence of the *ica*A and *ica*D, are similar to the results of other authors [33,38,39]. Although the majority of work reported the *ica*A and *ica*D to be nearly similar in incidence [40,41,42], our results do not confirm this relationship. Differences in the frequency of genes belonging to the same operon, as with the other authors [33,43,44], may be due to a mutation on *ica*A, resulting in differences in DNA sequences which may be the reason for the failed amplification of this genes. It is worth noting that difficulties in determining the presence of *ica*A and *ica*D genes were reported by Simojoki et al. [24] who excluded *ica*A and *ica*D results from their work. Similar to previous studies, the presence of *ica*A/*ica*D in the current study was not correlated with the strains’ capacity for biofilm formation. The results obtained in this, and other studies, emphasize the significance of the mechanisms of forming a biofilm independent of the *ica* operon.

Another gene which frequently occurs among the tested isolates of both *S. aureus* and CoNS was the *eno* gene which encodes the laminin-binding protein. In both groups, this gene was found in more than 50% isolates. However, many authors report that the *eno* gene was also detected in staphylococci that are not capable of forming a biofilm [24,37,45]. As regards the literature data, it can be assumed that the *eno* gene is not closely related to the adhesion capacities of staphylococci. However, this study demonstrated seven CoNS isolates with biofilm-forming capacities, which had this gene exclusively.

The staphylococcus’ potential for forming a biofilm is also determined by many other proteins (PIA-independent) that are independent of the *ica* operon [46]. A PIA-independent biofilm is associated with the presence of, inter alia, the *aap* protein responsible for the accumulation in the species *S. epidermidis*, or the *bap* protein, i.e., surface protein in both *S. epidermidis and S. aureus*. The frequency of the *bap* gene occurrence was 23.8% and 25% in CoNS strains and *S. aureus*, respectively. Even though the *bap* gene was the first to be isolated as a protein associated with the biofilm formation [45] many authors reported a much lower percentage or even failed to demonstrate its presence among the tested isolates originating from both food and clinical isolates [14,37,45,47]. In accordance with the literature data, the occurrence frequency of this gene among isolates is rather low [48]. The authors report the presence of the *bap* on the SaPIbov2 (Staphylococcal pathogenicity island bovine 2) exclusively in a small amount of *S. aureus* isolates originating mainly from the sub-clinical mastitis in cattle [15,49].

For CoNS strains, researchers detected another *bhp* protein which is a homologue of the *bap* gene. Due to the high similarity, it was assumed that the *bhp* is related to the CoNS strains’ capacity for biofilm formation [20]. Nevertheless, research into the relationship between this protein and the staphylococcal capacity for biofilm formation is still limited. Similar to the results obtained by other authors [50,51], the presence of *bhp* was not found in any of the tested isolates. The presence of the *aap* (accumulation-associated protein) was mainly demonstrated among the *S. epidermidis* species (60%) and in individual isolates of *S. haemolyticus*, *S. sciuri* and *S. simulans*. Srednik et al. [37] demonstrated the *aap* only among three *S. epidermidis* isolates, while a considerably higher percentage of strains with the *aap* present was obtained by Tremblay et al. [26] in their study where the majority of isolates had this gene. The high percentage of CoNS isolates with the *aap* among researchers might have been due to the various level of presence of particular species in the tested samples. The presence of *aap* had no significant effect on biofilm formation, which is especially significant since it occurred in combination with other genes. Similar observations were demonstrated by other authors [26,37]. The other genes (*fbe*, *atl*E, *emb*P) were present in a small percentage of isolates >25%, and they were mainly found in the following species: *S. epidermidis*, *S. simulans*, *S. haemolyticus* and *S. hominis*. Similar to the studies by other authors [8,26,37] the majority of these genes were found in combination with other genes. 

It is worth noting that the PCR technique may be insufficient to assess the genetic basis of biofilm production because presence/absence of a gene does not directly indicate that the encoded protein plays a role in the ability of staphylococci to form a biofilm. Therefore, further studies should target the use of advanced molecular biology techniques to better understand the genetic background of adhesion ability among *Staphylococcus* genus.

## 4. Materials and Methods 

### 4.1. Isolation of Staphylococci Strains

Thirty samples of raw cow milk were obtained from central Poland farms from healthy Holstein–Friesian cows. An amount of 10 mL was transferred to a flask containing 90 mL of buffered peptone water. The samples thus prepared were incubated for 24 h at 37 °C. After incubation, a loop full culture was streaked on Baird–Parker agar medium (Merck Millipore, Germany) for *Staphylococcus* spp. isolation. Typically, 1 to 5 different colonies were taken and streaked on Rabbit Plasma Fibrinogen—RPF agar plates (BioMérieux, Marcy-l’Étoile, France) to differentiate into coagulase-positive and coagulase-negative strains. Strain identification was performed using a MALDI (matrix-assisted laser desorption and ionization) instrument (Biomerieux). Firstly, all different colonies were streaked on Trypticase Soy Agar (Merck Millipore, Darmstadt, Germany) and incubated overnight at 37 °C. Next, a small amount of a colony was transferred to a metallic MALDI plate and then covered with 1 µL of CHCA matrix (α-Cyano-4-hydroxycinnamic acid) (BioMérieux). As recommended by the manufacturer, the *E. coli* ATCC 8739 strain was used as a calibrator. The prepared plate was placed in the measuring chamber of the device VITEK^®^ MS (BioMérieux), using MALDI (Matrix-Assisted Laser Desorption Ionization) technology, air was removed in order to obtain a vacuum and subjected to a laser beam. A dry sample in a matrix solution absorbs the energy from a laser beam resulting in ionization of the sample solution. All ions are accelerated to the same kinetic energy, enter the flight tube and are separated according to mass. Then, the algorithm associated with the Vitek MS system automatically identifies the tested organism by comparing the characteristics of the spectrum obtained (presence and absence of specific peaks) with those of the typical spectrum of each claimed species.

### 4.2. Detection of the Ability to Slime Production by Congo Red Agar (CRA) Method 

Slime production ability was determined using Congo Red Agar method [52]. CRA plates were prepared by adding 0.8 g of Congo red and 36 g of saccharose to 1 l of Brain Heart Infusion agar (Oxoid) [48]. The plates were incubated at 37 °C for 24 h. After incubation, the plates were stored at room temperature for 48 h. The ability to produce slime was interpreted according to their colony phenotypes. Black colony was considered as slime producers whereas bordeaux and red colonies were considered as non-producing strains.

### 4.3. Biofilm Forming Ability Detection by Microtiter Plate Method (MTP)

The ability to produce a biofilm was tested on 96-well, flat-bottomed, sterile polystyrene plates (Promed^®^) according to Stepanović et al. [53]. An amount of 200 μL of fresh sterile Trypticase Soy Broth was introduced into wells. A culture of strains (20 μL) with a density of 1 × 10^9^ cfu/mL was introduced into the broth wells in three repetitions. Negative controls were the wells without the addition of bacterial culture. The plates were incubated at 37 °C for 24 h. After incubation, the content of each well was gently pipetted and was then washed three times with 250 μL PBS (Phosphate Buffered Saline) buffer. The biofilm was then fixed for 15 min by adding 200 μL of 96% ethanol into each well. After that time, alcohol was poured in and plates were dried at room temperature for 20 min. Subsequently, the biofilms were stained for five minutes with a solution of crystal violet (200 μL). After staining, the plates were washed gently under a laminar stream of running water until the unbound dye was washed out from the wells. After drying the tiles at room temperature, 160 μL 33% (v/v) of glacial acetic acid was added. Absorbance at 570 nm wavelength was measured with spectrophotometric microplate reader Varioscan LUX (Thermo Scientific, San José, CA, USA). The optical density (ODs) for each test strain was determined based on the arithmetic mean of three repetitions taking measurements in 20 places of each well. The value obtained was compared with the cut-off value (ODc). Cut-off OD (ODc) is defined as three standard deviations above the mean OD of the negative control. Strains were interpreted as follows: non-biofilm producers (OD ≤ ODc); weak biofilm producers (ODc < OD ≤ 2× ODc); moderate biofilm producers (2× ODc < OD ≤ 4× ODc) and strong biofilm producers (4× ODc < OD).

### 4.4. Detection of Biofilm-Associated Genes

The following biofilm-associated genes: *ica*A, *ica*D, *eno*, *bap* among all staphylococci, and in addition *bhp*, *aap*, *fbe*, *emb*P, *atl*E among CoNS were amplified by PCR using specific primers (Table S1). The PCR products were visualized by electrophoresis on 1.5% agarose gels in 1×TBE (Tris-borate-EDTA) buffer stained by 0.5 μg/mL of ethidium bromide (0.5 mg/mL; Sigma-Aldrich Corp., St. Louis, MO, USA) and visualized using the system for the documentation and analysis of fluorescently stained gels G-BOX F3 (Syngene, Cambridge, UK).

### 4.5. Statistical Analysis

Statistical analyses were performed using STATISTICA 13.3 StatSoft^®^ (StatSoft, Inc., Tulsa, OK, USA) using the Spearman correlation test or the Mann–Whitney U-test. For the analyses, the significance level α = 0.05 was assumed.

## 5. Conclusions

The results of the study demonstrate that raw milk was a source of staphylococci capable of forming a biofilm. The formation of a biofilm by staphylococci isolated from raw milk may lead to the contamination of dairy products which, in turn, can cause food poisoning in consumers. The biofilm may result in economic losses of dairy plants and contribute to difficult-to-treat, recurrent udder inflammation (mastitis) in cattle. The obtained results confirm the adhesion properties of the *Staphylococcus* genus. The presence of potential biofilm-producing *S. aureus* and CoNS isolates in raw milk may present with severe human health and food factory challenges. The study underlines the validity of the biofilm problem faced by the food processing or hospital sectors. Understanding the mechanisms and genetic foundations responsible for the formation of biofilms is a significant element enabling the implementation of appropriate measures to prevent their formation.

## Figures and Tables

**Table 1:** Results of biofilm production ability and slime production.

Strains	Biofilm Production (MTP Method)			Slime Production (CRA Method)	
	Strong	Moderate	Weak	Positive	Negative
*S. haemolyticus* (n = 11)	8 (72.7%)	1 (9.1%)	2 (18.2%)	4 (36.4%)	7 (63.6%)
*S. simulans* (n = 8)	7 (87.5%)	-	1 (12.5%)	7 (87.5%)	1 (12.5%)
*S. epidermidis* (n = 6)	5 (83.3%)	1 (16.7%)	-	4 (66.7%)	2 (33.3%)
*S. warneri* (n = 5)	4 (80%)	-	1 (20%)	2 (40%)	3 (60%)
*S. chromogenes* (n = 4)	4 (100%)	-	-	2 (50%)	2 (50%)
*S. hominis* (n = 3)	3 (100%)	-	-	1 (33.3%)	2 (66.7%)
*S. sciuri* (n = 2)	2 (100%)	-	-	1 (50%)	1 (50%)
*S. saprophyticus* (n = 1)	1 (100%)	-	-	1 (100%)	-
*S. capitis* (n = 1)	1 (100%)	-	-	-	1 (100%)
*S. xylosus* (n = 1)	1 (100%)	-	-	1 (100%)	-
Total CoNS (n = 42)	36 (85.7%)	2 (4.8%)	4 (9.5%)	23 (54.8%)	19 (45.2%)
Slime producing CoNS (n = 23)	20 (87%)	1 (4.3%)	2 (8.7%)	-	-
No slime-producing CoNS (n = 19)	16 (84.2%)	1 (5.3%)	2 (10.5%)	-	-
*S. aureus* (n = 12)	7 (58.3%)	1 (8.3%)	4 (33.3%)	5 (41.7%)	7 (58.3%)
Slime producing *S. aureus* (n = 5)	4 (80%)	0	1 (20%)	-	-
No slime-producing *S. aureus* (n = 7)	3 (42.9%)	1 (14.2%)	3 (42.9%)	-	-
All staphylococci (n = 54)	43 (79.6%)	3 (5.6%)	8 (14.8%)	28 (51.9%)	26 (48.1%)

**Table 2:** Biofilm-associated genes among staphylococci.

	Biofilm-Associated Gene								
Strains	*ica*A	*ica*D	*bap*	*eno*	*aap*	*fbe*	*bhp*	*emb*P	*atl*E
*S. haemolyticus* (n = 11)	5 (45.5%)	2 (18.2%)	3 (27.3%)	7 (63.6%)	3 (27.3%)	2 18,2%)	-	-	-
*S. simulans* (n = 8)	4 (50%)	2 (25%)	1 (12.5%)	2 (25%)	3 (37.5%)	-	-	2 (25%)	5 (62.5%)
*S. epidermidis* (n = 6)	-	2 (33.3%)	3 (50%)	4 (66.7%)	5 (60%)	1 (16.7%)	-	4 (66.7%)	4 (66.7%)
*S. warneri* (n = 5)	1 (20%)	1 (20%)	1 (20%)	3 (60%)	-	-	-	-	-
*S. chromogenes* (n = 4)	1 (25%)	-	-	2 (50%)	-	-	-	-	-
*S. hominis* (n = 3)	1 (33.3%)	-	-	-	-	-	-	-	1 (33.3%)
*S. sciuri* (n = 2)	1 (50%)	-	2 (100%)	2 (100%)	1 (50%)	-	-	1 (50%)	-
*S. sapropythicus* (n = 1)	-	1 (100%)	-	-	-	-	-	-	-
*S. capitis* (n = 1)	-	1 (100%)	-	-	-	-	-	-	-
*S. xylosus* (n = 1)	-	-	-	-	-	-	-	-	-
Total CoNS (n = 42)	12 (31.0%)	9 (21.4%)	10 (23.8%)	20 (47.6%)	12 (28.6%)	3 (7.1%)	-	7 (16.7%)	10 (23.8%)
*S. aureus* (n = 12)	-	12 (100%)	3 (25%)	7 (58.3%)	NA	NA	NA	NA	NA
All staphylococci (n = 54)	13 (24.1%)	21 (38.9%)	13 (24.1%)	27 (50.0%)	-	-	-	-	-

NA—not applicable.

**Table 3:** Association between results of Microtiter Plate Method and amplification biofilm-related genes in *S. aureus* and coagulase-negative (CoNS) isolates.

Species	MTP Method	No.	Number (%) Strains with								
			*ica*A	*ica*D	*bap*	*eno*	*aap*	*fbe*	*bhp*	*emb*P	*atl*E
*S. aureus* (n = 12)	Strong	7	0	7 (100%)	2 (28.6%)	4 (57.1%)	-	-	-	-	-
	Moderate	1	0	1 (100%)	0	1 (100%)	-	-	-	-	-
	Weak	4	0	4 (100%)	1 (25%)	2 (50%)	-	-	-	-	-
CoNS (n = 42)	Strong	36	10 (27.8%)	7 (19.4%)	10 (27.8%)	17 (47.2%)	9 (25%)	3 (8.3%)	0	6 (16,7%)	8 (22.2%)
	Moderate	2	0	1 (50%)	0	0	2 (100%)	0	0	1 (50%)	1 (50%)
	Weak	4	3 (75%)	1 (25%)	0	3 (75%)	1 (25%)	0	0	0	1 (25%)

## Supplementary Materials

The following are available online at https://www.mdpi.com/2076-0817/9/8/654/s1, Table S1: List of primer sequences used in PCR.
