# Supplementary material for: Biofilm Formation Ability and Presence of Adhesion Genes among Coagulase-Negative and Coagulase-Positive Staphylococci Isolates from Raw Cow’s Milk
Source: Pathogens. 2020 Aug 14;9(8):654. doi: 10.3390/pathogens9080654 (PMC7460418; doi:10.3390/pathogens9080654)
Supplement: Supplementary file 1 [file pathogens-09-00654-s001.pdf]

pathogens

MDPI

Supplementary Materials

Table 1. List of primer sequences used in PCR reactions.

Gene	Primer Sequence	Annealing Temp. (°C)	Amplicon Size (bp)	References
***ica*A** (intercellular adhesion gene)	F: TCTCTTGCAGGAGCAATCAA<br>R: TCAGGCACTAACATCCAGCA	55,5	188	[1]
***ica*D** (intercellular adhesion gene)	F: ATGGTCAAGCCCAGACAGAG<br>R: CGTGTTTTCAACATTTAATGCAA<br>R: GCTCTTGTAAGACCATTTTCTTCAC	55,5	199	[1]
bap (biofilm associated protein)	F: CCCTATATCGAAGGTGTAGAATTG<br>R: GCTGTTGAAGTTAATACTGTACCTGC	60	971	[2]
eno (laminin binding protein)	F: ACGTGCAGCAGCTGACT<br>R: CAACAGCATCTTCAGTACCTTC	55	302	[3]
aap (accumulation associated protein)	F: AAACGGTGGTATCTTACGTGAA<br>R: CAATGTTGCACCATCTAAATCAGCT	60	466	[4]
bhp (bap homologue protein)	F: CTACAAGTTCAGGTCAAGGACAAGG<br>R: GCGTCGGCGTATATCCTTCAG	60	1583	[4]
fbe (fibrinogen binding protein)	F: CTACAAGTTCAGGTCAAGGACAAGG<br>R: GCGTCGGCGTATATCCTTCAG	60	495	[5]
***emb*P** (extracellular matrix binding protein)	F: AGCGGTACAAATGTCAAT<br>R: AGAAGTGCTCTAGCATCATCC	57	455	[5]
atl*E** (autolysin E***)	F: CAACTGCTCAACCGAGAACA<br>R: TTTGTAGATGTTGTGCCCCA	55	682	[5]

References

1. Arciola, C.R.; Baldassarri, L.; Montanaro, L. Presence of icaA and icaD genes and slime production in a collection of Staphylococcal strains from catheter-associated infections. *J. Clin. Microbiol.* **2001**, *39*, 2151–2156.
2. Cucarella C., Solano C., Valle J., Amorena B., Lasa I., P.J.R. Bap, a Staphylococcus aureus Surface Protein Involved in Biofilm Formation Staphylococcus aureus Surface Protein Involved in Biofilm Formation. *Microbiology* **2001**, *183*, 2888–2896.
3. Tristan, A.; Ying, L.; Bes, M.; Etienne, J.; Vandenesch, F.; Lina, G. Use of multiplex PCR to identify Staphylococcus aureus adhesins involved in human hematogenous infections. *J. Clin. Microbiol.* **2003**, *41*, 4465–4467.
4. Rohde, H.; Burdelski, C.; Bartscht, K.; Hussain, M.; Buck, F.; Horstkotte, M.A.; Knobloch, J.K.M.; Heilmann, C.; Herrmann, M.; Mack, D. Induction of Staphylococcus epidermidis biofilm formation via proteolytic processing of the accumulation-associated protein by staphylococcal and host proteases. *Mol. Microbiol.* **2005**, *55*, 1883–1895.
5. Rohde, H.; Burandt, E.C.; Siemssen, N.; Frommelt, L.; Burdelski, C.; Wurster, S.; Scherpe, S.; Davies, A.P.; Harris, L.G.; Horstkotte, M.A.; et al. Polysaccharide intercellular adhesin or protein factors in biofilm accumulation of Staphylococcus epidermidis and Staphylococcus aureus isolated from prosthetic hip and knee joint infections. *Biomaterials* **2007**, *28*, 1711–1720.

© 2020 by the authors. Submitted for possible open access publication under the terms and conditions of the Creative Commons Attribution (CC BY) license (http://creativecommons.org/licenses/by/4.0/).
